# Chemical Profile and Antibacterial Activity of a Novel Spanish Propolis with New Polyphenols also Found in Olive Oil and High Amounts of Flavonoids

**DOI:** 10.3390/molecules25153318

**Published:** 2020-07-22

**Authors:** María Coronada Fernández-Calderón, María Luisa Navarro-Pérez, María Teresa Blanco-Roca, Carolina Gómez-Navia, Ciro Pérez-Giraldo, Virgina Vadillo-Rodríguez

**Affiliations:** 1 Networking Biomedical Research Centre on Bioengineering, Biomaterials and Nanomedicine (CIBER-BBN), 06006 Badajoz, Spain; matere@unex.es (M.T.B.-R.); giraldo@unex.es (C.P.-G.); vvadillo@unex.es (V.V.-R.); 2 Department of Biomedical Science, Area of Microbiology, University of Extremadura, 06006 Badajoz, Spain; marisanav@unex.es (M.L.N.-P.); cgomezna@alumnos.unex.es (C.G.-N.); 3 University Institute of Extremadura Sanity Research (INUBE), 06006 Badajoz, Spain; 4 Department of Applied Physics, University of Extremadura, 06006 Badajoz, Spain

**Keywords:** antibacterial activity, flavonoids, olive oil, phenolic compounds, propolis, *Staphylococcus*

## Abstract

Propolis is a natural product obtained from hives. Its chemical composition varies depending on the flora of its surroundings, but nevertheless, common for all types of propolis, they all exhibit remarkable biological activities. The aim of this study was to investigate the chemical composition and antimicrobial activity of a novel Spanish Ethanolic Extract of Propolis (SEEP). It was found that this new SEEP contains high amounts of polyphenols (205 ± 34 mg GAE/g), with unusually more than half of this of the flavonoid class (127 ± 19 mg QE/g). Moreover, a detailed analysis of its chemical composition revealed the presence of olive oil compounds (Vanillic acid, 1-Acetoxypinoresinol, *p*-HPEA-EA and 3,4-DHPEA-EDA) never detected before in propolis samples. Additionally, relatively high amounts of ferulic acid and quercetin were distinguished, both known for their important therapeutic benefits. Regarding the antimicrobial properties of SEEP, the minimal inhibitory and bactericidal concentrations (MIC and MBC) against *Staphylococcus epidermidis* strains were found at the concentrations of 240 and 480 µg/mL, respectively. Importantly, subinhibitory concentrations were also found to significantly decrease bacterial growth. Therefore, the results presented here uncover a new type of propolis rich in flavonoids with promising potential uses in different areas of human health.

## 1. Introduction

In recent years, there has been a renewed interest in the propolis composition and biological activity. Propolis is a natural substance collected by honeybees from buds and exudates of certain plants and trees, mixed with pollen and salivary enzymes secreted by the bees themselves. It is used by these flying insects to seal the holes in their hives, smooth out the internal walls, exclude draught and, most importantly, protect their hive from intruders [1]. It has been used in folk medicine for centuries, and modern science has revealed substantial evidence indicating that propolis has antibacterial, antifungal, antiviral, antioxidant, anti-inflammatory, antitumor and immunomodulating properties [2,3]. Particularly, due to its antibacterial and antifungal properties, it has recently attracted much attention as an alternative to minimize the spread of antibiotic resistance in pathogenic bacteria in the medical field.

The composition of ethanolic extracts of propolis, the form in which it is most commonly used, differs greatly due to variations in geographical and botanical origin [4]. This aspect clearly hinders propolis standardization but, nevertheless, certain flavonoids and esters of phenolic acids are generally regarded to be responsible for the antimicrobial activity of propolis [5]. Additionally, it is worth noting that phenolic compounds are among the most potent and therapeutically useful bioactive substances providing health benefits associated with a lower risk of chronic and degenerative diseases. Many of these effects are attributed to antioxidant activity and the blocking of free radicals [6].

Propolis chemical composition depends on geographical location, but it also varies with seasonality, illumination, altitude, bee species, extraction method, parallel activity in the area and food availability [7]. It is generally accepted that propolis from temperature climatic zones (i.e., Central Europe, North America and non-tropical regions of Asia) originates mainly from the bud exudates of *Populus* species and their hybrids. These exudates are typically rich in chrysin, pinocenbrim, pinobanksin-acetate, galagin and caffeic and ferulic acids, all phenolic compounds reported to possess antimicrobial activities [8]. Propolis from tropical climatic zones (i.e., Brazilian propolis), however, is typically rich in prenylatedbenzophenons, dipertenes and flavonoids [9]. Moreover, in the last decade, Popova et al. described a new type of European propolis, the Mediterranean type (collected in certain locations of Greece and Greek islands), which contains mainly diterpenes and almost no phenolic compounds [10]. Its botanical origin is yet unidentified, but on the basis of the identified diterpenes, the source plant was suggested to be some conifer species of the *Cupressaceae* family. Despite differences in the chemical composition, all types of propolis analyzed exhibit, to a lesser or greater extent, antimicrobial activities. It is evident that bees have the ability to collect from their environment natural antimicrobial agents to protect their hives. For this reason, propolis chemical diversity has the potential to provide valuable leads, and it remains essential to link propolis antimicrobial properties to a detailed investigation of its chemical composition (and botanical sources).

The efficacy of propolis in different protocols in vitro and in vivo suggests its antimicrobial and therapeutic properties [11,12], but before establishing a strategy using this bee product, it is necessary to specify its chemical nature to determine the principal microbiologically active molecules. In general, the antibacterial activity of propolis is connected with both the direct action on the microorganisms and with the stimulation of the immune sisyten resulting in activation of natural defences of the organisms [11]. Interestedly, it is generally observed that the antimicrobial activity of propolis is higher in Gram-positive than Gram-negative bacteria. For the range of propolis types mentioned about, i.e., the propolis from temeperature climate zones, tropical climate zones and the new European type, the average MIC values reported range from 117 to 1840 μg/mL for Gram-positive and from 34 to 5000 μg/mL for Gram-negative cells [13]. Regarding the Gram-positive *S. epidermidis*, one of the least tested bacteria in relation to the antibacterial activity of propolis, MIC valures from 8 to 1135 µg/mL have been registered [13].

In the present study, a new type of propolis from Extremadura in the Southwest of Spain has been examined. In the literature, few data can be found about the chemical composition of Spanish propolis extracts [14,15,16,17,18,19] or their antimicrobial activity [20], and no data at all about propolis from Herrera del Duque, the exact location of collection of the propolis samples here evaluated. Its chemical profile has been obtained from Liquid chromatography–mass spectrometry (LC-MS) and high-resolution gas chromatography coupled to mass spectrometry (GC–MS). The antimicrobial activity of this new Spanish propolis has been tested against four different strains of *Staphylococcus epidermidis*, microorganism of the skin and mucous microbiota of humans and animals that serve as a reservoir of resistance genes [21].

## 2. Results and Discussion

### 2.1. Determination of Polyphenol and Total Flavonoid Content

Investigations have revealed that polyphenols play a key role to prevent bacterial infections and various diseases, like hypercholesterolemia, hyperglycemia, hyperlipidemia, and cancer insurgence [22,23,24,25]. Therefore, for propolis samples, quantification of the total polyphenol content is used as a measure for the amount of active principles.

For the SEEP studied, the total content of phenolic compounds (TPC) was 205 ± 34 mg GAE/g of SPEE. The results also showed that more than half of these are compounds of the flavonoid class (127 ± 19 mg QE/g of SEEP). Propolis samples from Spain typically show high amounts of polyphenols, with values that range from 31.4 to 364 mg GAE/g and 200 to 300 mg GAE/g from samples collected at East Andalusia [18] and Basque Country [17], respectively, but shows low TFC/TPC ratios (0.39–0.47). The TFC/TPC ratio here found (~0.6) is the higher ever reported, both in Spain and almost around the world.

In particular, from more than twenty studies reviewed from around the world, only eight of them stated higher concentrations of TPC than that found in the present study, and only one of them showed a slightly higher TFC/TPC ratio (i.e., ~0.7), with 176 mg/g of flavonoid versus 242 mg/g of polyphenols [26]. Interestingly, previous research has shown that the polyphenolic and flavonoid contents of propolis increase during the warmest period of the year. The particular geometrical location of Herrera del Duque, surrounded by mountain ranges, grants to the region an average annual temperature of 16.3 °C, which is higher than other European regions of Greece such as Thessaloniki, or Italy, Germany or Hungary [27], which may explain the high flavonoid content obtained here.

### 2.2. LC-MS and GS-MS

**Figure 1:**
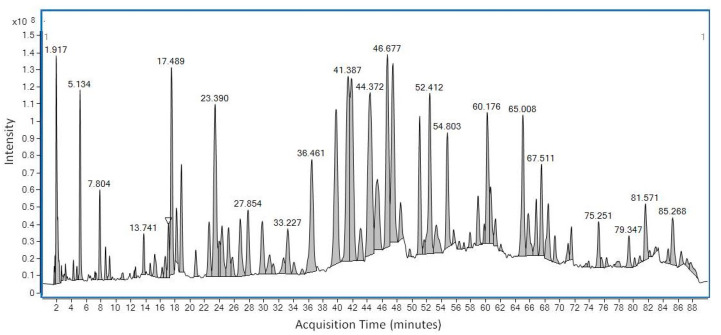
LC-MS chromatogram of Spanish Ethanolic Extract of Propolis (SEEP) showing the major and minor organic peaks found in the sample.

**Figure 2:**
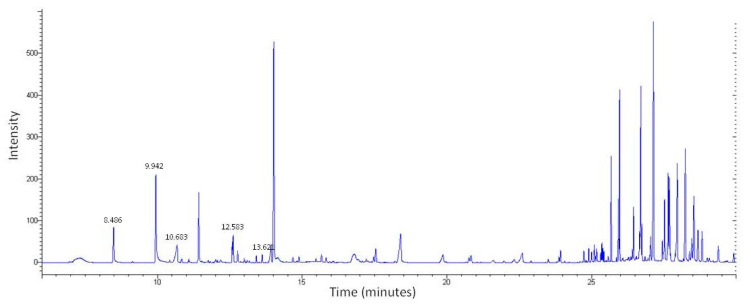
GC–MS profile of SEEP showing the compounds compiled in Table 1.

LC-MS was first used to determine the chemical profile of SEEP. The chromatogram obtained showed a complex chemical composition with various peaks at different retention times (Figure 1). A total of 56 peaks were clearly identified and classified according to the main classes of polyphenols: flavonoids, phenolic acids, ligands, etc., (Table 1). The volatiles of SEEP samples were also analyzed by GS-MS. It is important to mention that most of the constituents of propolis are relatively polar (flavonoids, phenolic acids and their esters, etc.), and silylation is necessary to increase their volatility and enable GC analysis. The results of the GC–MS profile are shown in Figure 2, and the compounds identified are shown in Table 1.

**Figure 3:**
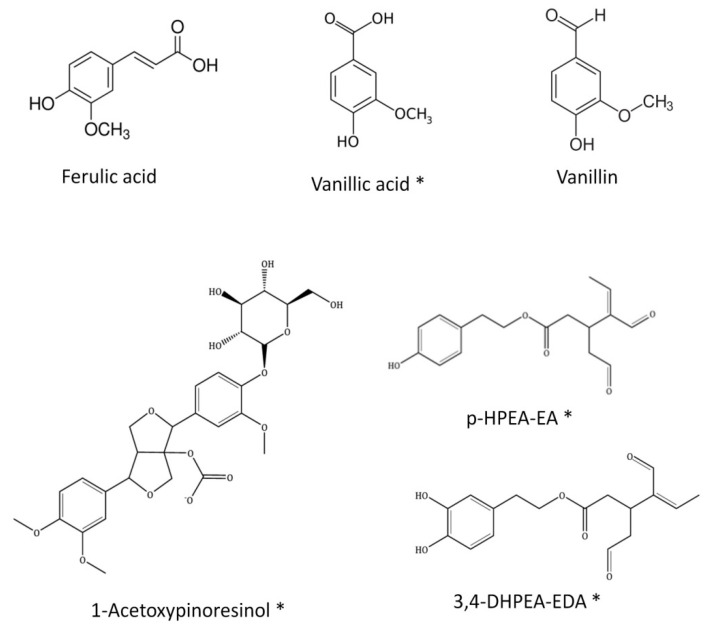
Chemical structure of the compounds identified in SEEP previously found in olive oils. (*) Compounds indentified for the first time in a propolis sample.

Similar to other types of propolis samples, flavonoids, phenolic acids, lignans and a few other different kinds of polyphenols have also been identified in SEEP [8,28]. Remarkably, Ferulic acid, Vanillic acid, 1-Acetoxypinoresinol (lignan), *p*-HPEA-EA (ligstroside derivative) and 3,4-DHPEA-EA (oleuperin derivative), and Vanillin (phenolic aldehyde) are all found in commercial virgin olive oils [29,30,31,32], together with *p*-coumaric acid, caffeic acid, luteolin, apigenin and a few other minor phenolic compounds. All the aforementioned compounds are present in SEEP (highlighted in bold in Table 1), adding an exceptional value to this new Spanish propolis. Exceptionally, at least four of these compounds, never before reported in propolis, could be identified: 1-Acetoxypinoresinol, 3.4-DHPEAEDA, *p*-HPEA-EA and Vanillic acid. The chemical structure of these compounds is shown in Figure 3.

It has been suggested that high concentrations of phenolic compounds in olive oil may contribute to the healthy action of the Mediterranean diet because they exhibit protective effects against neuro-degenerative and cardiovascular diseases and even show antiproliferative effects, contributing to protect the organism against oxidative damage [33,34] and infectious disease [35].

The analysis of propolis of various geographic areas has shown that European, Chinese and Argentinean propolis [8] are characterized by the presence of secondary metabolites characteristic for the buds of *Populus* spp.: pinocembrin, pinobanksin and its acetate, prenyl esters of caffeic and ferulic acids. Previous research has also shown that Greek and Mediterranean propolis in general are not very rich in polyphenols and considered rather poor in flavonoids, but are very rich in diterpenes, characterized by the presence of a substantial amount of communic, cupressic, and isocupressic acids and totarol, similar to that previously found in propolis from Brazil [36]. Their botanical origin is yet unidentified, but on the basis of the diterpenic profile, the source plant has been suggested to be some conifer species of the *Cupressaceae* family. Propolis from the western countries of the Mediterranean basin and Portugal typically show a heterogeneous chemical composition. Nevertheless, these samples are divided into two different groups: the first characterized by relatively high amounts of phenol acids and their derivatives and flavonoids of poplar-type, and a second one in which diterpenes dominate [37]. Interestingly, SEEP, also a Mediterranean-type propolis, does not contain dipertenes or the typical pattern of “poplar type” propolis; it is characterized by a high amount of polyphenol and flavonoids, including *p*-coumaric, caffeic and ferulic acids and phenolic compounds found in extra virgin olive oil.

These results can be useful in confirming that the plant sources of propolis determine its composition. In previous studies, it was noted that even in Europe, where propolis is believed to be very well studied, there could be surprises concerning the plant origin and chemical composition of bee glue [38]. In the collection area (Herrera del Duque, Extremadura) of the SEEP, the most extended ecosystem is the Dehesa, with a significant number of hectares dedicated to olive trees (*Olea europaea*).

### 2.3. Quantification of Marker and Metal Compounds

The dry weight of propolis obtained per mL of ethanolic extract was 61.5 mg/mL. Moreover, calibration curves were constructed for three standards: vanillic acid, the new compound identified in SEEP and in olive oil, trans-ferulic acid, as a representative of an olive oil component, and quercetin, the most commonly used flavonoid as standard in calibration curves. Regression analysis was employed to determine the linearity of the calibration graphs and the calculated equations are reported in Table 2. It was found that the vanillic acid peak represents a concentration of 5.2 μg/mL (equivalent to 0.084 mg/g propolis dry) and the trans-ferrulic acid and quercetin peaks represented 250 μg/mL (eqv 4.065 mg/g) and 23.5 μg/mL (eqv 0.382 mg/g), respectively.

The detected concentration of vanillic acid, identified for the first time in a propolis sample, increases the value of this new SEEP by its protective capacities against several diseases associated with oxidative deterioration [39]. It has also been reported that it can selectively inhibit the growth of pathogenic bacteria without affecting the viability of probiotics [40]. Moreoever, the measured concentration of ferulic acid (4-hydroxy-3-methoxy-cinnamic acid) is higher than that found, for example, in Russian [28] or Greek propolis [41], and within in the concentrations range found in other Spanish propolis [18]. Importantly, the ferulic acid is a phenolic compound with important antioxidant effects which may offer beneficial effects against cancer, cardiovascular disease, diabetes and Alzheimer’s disease [42]. Quercetin, on the other hand, presents antioxidant and anti-inflammatory activities and prevents cancer [43]. The concentration of this compound in the new SEEP is also higher than the found in Greek propolis [41]. Interestingly, propolis from Argentina contains similar concentrations of ferulic acid (from 0.51 to 6.42 mg/g) and quercetin (from 0 to 2.84 mg/g) to SEEP [44].

Finally, it is also known that flavonoids are chelators of trace metals and exhibit, for example, antioxidant properties by joining the transition metals [45]. Likewise, metals could form a strong ligand complex with flavonoids and enhance the bactericidal activity of these complexes. Even though the metal composition of propolis samples has been hardly studied, the toxicity of propolis has been occasionally attributed to its heavy metal content [28]. The results here obtained through X-ray fluorescence spectrometry revealed that SEEP contains negligible traces of K, P, Zn and Cu (Table 3), and toxic metal compounds, such as Pb, Cr or Cd, were not detected at all.

### 2.4. Antimicrobial Activity

All propolis samples studied up to date showed, to a lesser or greater extent, antimicrobial activity independently from their geographic origin or chemical consistency. The MICs and MBCs estimated in the current study for the selected *S. epidermidis* strains, one of the least tested bacteria in relation to the antibacterial activity of propolis [13], were 0.39% (240 µg/mL) and 0.78% (480 µg/mL), respectively. The biological activity of SEEP detected was not influenced by the presence of ethanol in the propolis solutions. The MIC and MBC of the alimentary ethanol in which the propolis was dissolved were above 6%. The fact that MBC’s values are so near to MIC’s is indicative of the good bactericidal capacity of SEEP.

A survey of the literature on the antimicrobial activity of the different types of propolis against *S. epidermidis* (Table 4) reveals that, around the world, only a couple samples showed greater activity against this strain than SEEP. These particular samples, originally from Greece and Turkey, delivered mean MIC values for *S. epidermidis* of 50 and 32 µg/mL, respectively [46,47]. It should be noted, however, that the specific experimental methodology used in these studies, i.e., the microdilution in broth, is likely to overestimate the MIC values reported (as mentioned earlier, the colour of propolis readily interferes with the spectrophotometer readings). On the other hand, among the Spanish ethanolics extracts of propolis, none have been tested against *S. epidermidis* to date. Only one was tested against *S. aureus*, and despite this sample showing higher TPC values than the ones reported here, the MIC values registered ranged from 600 to 1.300 µg/mL [20].

It is also noteworthy that the methodology used in the present study was the agar dilution method (the colour of propolis would interfer with the values obtained by spectrophotometry). This methodology, the agar dilution method, has also been used by a few groups studying the antibacterial activities of different extracts against *S. epidermidis* [48,49,50,51]. In none of these studies was the measured antibacterial activity better than the found with SEEP.

The high amounts of phenolic compounds found in propolis have been associated with its antimicrobial activity [20]. Particularly, flavonoid, aromatic acids and esters are generally regarded as responsible for the antimicrobial activity of propolis. Galangin, chrysin, pinocembrin and pinostropin have been recognized as the most effective flavonoid agents against bacteria [36,44,52]. Interestingly, a great amount of pinocembrin (~39% of the total identified flavonoids), a compound not identified in the present work, was detected by Volpi and Bergonzini in a Spanish propolis sample [16]. Ferulic and caffeic acid, present in high amounts in the propolis samples analyzed from two different locations of Spain [14], have also been reported to contribute to the bactericidal action of propolis. In Brazilian propolis, however, isoflavonoid, (as neovestitol and vestitol) [53] and their derivates (as medicarpin) have been made responsible for the antibacterial activity of these samples [54].

The SEEP here studied, however, does not contain any of the aforementioned flavonoid agents regarded as responsible for the antibacterial properties of propolis, and yet shows substantial antimicrobial activity. This finding clearly indicates that different substances in SEEP are responsible for its biological activity. Interestingly, some of the unique compounds found in SEEP, i.e., Vanillic acid, Vanillin, Ferulic acid and Cinnamic acid, which have also been identified in olive oil, are widely known to display antimicrobial activities [35]. Additionally, a recent study on the bioactivity of olive oil samples revealed that the dialdehydic form of decarboxymethyl oleuropein-aglycone (3,4-DHPEA-EDA) and ligstroside aglycone (*p*-HPEA-EA), both tyrosols also present in SEEP, were the only two substances that statistically correlated with enhanced antimicrobial activity [55]. Thus, these compounds, detected for the first time in SEEP, could be responsible for the high bactericidal capacity uncovered for this new propolis.

**Figure 4:**
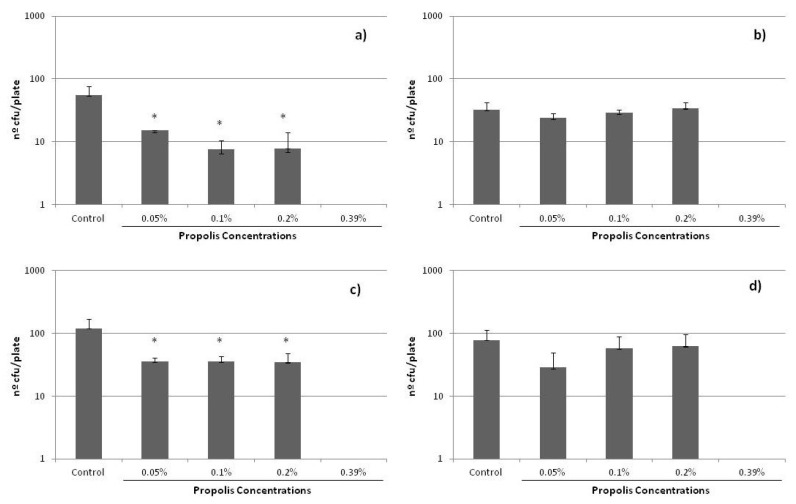
Effect of sub-MICs of SEEP on colonies forming on solid agar plates for the *S. epidermidis* strains studied: (**a**) ATCC 12228, (**b**) HAM 892, (**c**) ATCC 35983, (**d**) ATCC 35984. (*) indicates statistically significant differences (i.e., *p* < 0.05) with respect to the control samples.

Finally, it is important to recall that, in the course of antibiotic treatment, the concentrations inside many tissues may be lower than the MIC. These sub-inhibitory concentrations (sub-MICs) do not kill bacteria, but potentially modify their physical and chemical surface characteristics and, consequently, the possible expression of some virulence factors. It is for this reason that the influence of sub-MICs concentrations of propolis on bacterial growth was also analyzed in the present study. It is important to note that no data have been previously reported on this matter, significant in determining the complete activity of any propolis sample. Figure 4 shows the results obtained, i.e., the growth of the cells in plate (number of cfu/plate) after 24 h of incubation with the different sub-MICs of propolis evalutated. A decrease in growth was found for all the strains, which was especially significant for *S. epidermidis* ATCC 12228 and ATCC 35983, even at the lowest sub-MIC concentration studied, i.e., 0.05%.

The antimicrobial properties of propolis are related to the possible synergistic effect of its components, which may differ depending on its origin. In addition, for Brazilian propolis, for instance, it has also been demonstrated that the time or season of harvesting quantitatively affects its chemical composition and antimicrobial activity [63]. It would be thus interesting to evaluate the stability of SEEP chemical composition and antimicrobial activity over the years and as a function of the time of collection, i.e., seasonality. Nevertheless, this new Spanish propolis seems a promising source of new bioactive compounds first reported in propolis; remarkably, these compounds, also found in commercial olive oil, possess excellent biological and pharmaceutical properties known to promote human health. Thus, SEEP offers a new research pathway of interest in the field of pharmaceutical industries for obtaining natural substances with antibacterial activity alone or synergistically. These data, together with the widespread appearance of antibiotic resistance and the increasing interest towards natural products, suggest further studies for the best comprehension of which propolis compounds are involved in the antibacterial activity.

## 3. Materials and Methods

### 3.1. Propolis Samples Preparation

The Spanish ethanolic extract of propolis (SEEP) was provided by “La Virgen de Extremadura” (Artesanos Virgen de Extremadura, S.L, Badajoz, Spain). This extract is collected in the region of Extremadura, in the southwest of Spain, particularly at the location of Herrera del Duque. SEEP was produced by mixing the propolis gathered (twice, in spring and autumn) within each year. The ethanolic extract was filtered with a 0.20 µm syringe filter (Millipore, Merck, Darmstandt, Germany) and stored at 4 °C until use. For antibacterial activity assays, serial twofold dilutions of SEEP and its solvent (70° food alcohol) were prepared in TSB (i.e., Trypticase Soy Broth from BBL, Becton Dickinson and Company, Sparks, NV, USA) to obtain the final concentrations of 12% to 0.05%. In order to ensure the dry propolis amount in the different solutions, an aliquot of the original ethanolic extract was left to dry in the Pasteur Heraus electronic oven (C.R. Maré, S.A., Barcelona, Spain) for 3 h at 50 °C, and then weighed on the Sartorius precision analytical balance (model CP64).

### 3.2. Determination of Total Polyphenol and Flavonoid Contents

The total polyphenol content (TPC) of SEEP was determined using the Folin–Ciocalteu colorimetric method described by Frozza et al. [64], with some modifications. Briefly, 100 µL of SEEP was mixed with 500 µL of Folin-Ciocalteu and, after 5 min in the dark, 400 µL of 7.5% sodium carbonate (Na_2_CO_3_) was added. The absorbance of the reaction mixture was measured at 765 nm using a spectrophotometer (Helios epsilon, Thermo Scientific, Waltham, MA, USA) after 30 min of incubation at room temperature in the dark. Gallic acid standard solutions (0–250 mg/L) were used for the calibration curve. The TPC was expressed as mg of gallic acid equivalents (GAE) per gram of SEEP.

The total flavonoid content (TFC) of SEEP was determined according to the method described by Campos et al. [65], with minor modifications. For this purpose, 0.5 mL of SEEP was mixed with 4.5 mL of 2% aluminum chloride hexahydrate (AlCl_3_ 6H_2_O_2_) in methanol. The absorbance was read at 415 nm after 30 min of incubation at room temperature in the dark, using a spectrophotometer. Quercetin (0–5 mg/mL) was used as a standard to produce a calibration curve. The flavonoid content was expressed as mg of quercetin equivalents (QE) per gram of SEEP. The values of polyphenol and flavonoids are reported as mean ± standard derivations (SD) of three independent determinations.

### 3.3. Liquid Chromatography Mass Spectrometry Analysis (LC–MS)

The SEEP samples (1 μL) were introduced into a LC-DAD-MS system, an HPLC (Agilent 1200, Arcade, NY, USA) equipped with a qTOF mass analyzer 6520 Accurate Mass qTOF LC/MS. A Zorbax Eclipse PlusC 18 analytical column (100 × 4.6 mm, 3.5 µm Agilent, NY, USA) was used for separation at a flow rate of 0.5 mL/min. The column was maintained at 30 °C and the flow rate split 5:1 before the dual ESI source. The separation was performed by means of a linear gradient elution (eluent A, 0.1% formic acid; eluent B, acetonitrile). The gradient was as follows: initial 20% B, 20–30% B in 10 min, 30–40% B in 40 min, 40–60% B in 20 min, 60–90% B in 20 min and 90% B for 5 min and 20% B in 1 min and finally 20% B in 4 min, with a total time of 100 min. The mass spectrometer was operated in the negative full- scan mode in the range 100–1700 Da. LC–MS was carried out with capillary and fragment at voltages set to 3500 and 150 V, respectively, and a desolvation temperature of 350 °C. Data were acquired using the MassHunter Workstation Software v B0.6.01 (Agilent Technologies, Santa Clara, CA, USA) UV data were obtained at 254 and 280 nm.

The identification of the compounds was performed using commercial libraries (i.e., the polyphenol database of http://phenol-explorer.eu) by comparison of their mass spectra and retention times with reference compounds. In the cases where the was a lack of corresponding reference compounds, the structures were proposed on the basis of their general fragmentation and using the reference literature spectra, where possible.

### 3.4. Gas Chromatography Mass Spectrometry Analysis (GC–MS)

The GC-MS SEEP analys was performed as previously described Alencar et al. [66], with some modifications. Aliquots of 400 µL of SEEP were placed into glass vials. Samples were analyzed by Bruker Scion GC–TQ-MS using a 30 m × 0.25 mm i.d, 0.25 µm film. A HP-5MS column installed in a 456GC Bruker chromatograph instrument interfaced with a SCION TQ (Triple Quadrupole mass detector) was operated in scan mode (*m*/*z* 45–450). The GC-MS analysis was temperature programmed from 50 °C (0.3 min hold) to 285 °C (15 min hold) at 6 °C/min. Samples were injected with a Combi PAL autoinjector using a splitless injection technique (0.6 µL injection volume). Carrier gas (He) flow was set at 1.0 mL/min. The compounds were identified by the contrast of the acquired spectra (acquisition range 45–450 *m*/*z*) with the NIST spectrum library.

### 3.5. Quantification of Marker Compounds

The quantification of marker compounds was conducted following the method described by Ambi et al. [28], with some modifications. The standards compounds (Vanillic acid, *trans*-Ferulic acid and Quercetin) were purchased from Sigma (Sigma, St. Louis, MO, USA.) and prepared by dissolving the standard in HPLC grade ethanol to make concentrations of 5, 10, 25, 50 and 300 μg/mL. Later, 1 μL of each standard was injected into the LC-MS, under the same experimental conditions as described above. The calibration graphs were constructed by plotting the mean peak intensity against concentration. The linearity was investigated by generating the regression plots by the least squares method and determining the correlation coefficient (*R*^2^). The limit of detection (LOD) and the limit of quantification (LOQ) was obtained from the y-intercept standard deviation (*S_b_*) and the slope (*m*) of the calibration curve, thus LOD = 3 × *S_b_*/*m* and LOQ = 10 × *S_b_/m.*

### 3.6. Detection of Metal Ions/Complexes in SPEE

The metal ions in SEEP were determined by Wavelength dispersive X ray fluorescence (WDXRF, model S8 Tiger, Bruker Corporation, Billerica, MA, USA). The sample was measured in a liquid system deposited on the sample holder of the system (prolene 4 µm thickness, transparent to R-X). Approximately 8 g of sample was measured with a 28 mm mask in best acquisition mode with 14 min of capture time. The analysis of the data was done using standard methods.

### 3.7. Antibacterial Activity of Propolis

Four strains of *Staphylococcus epidermidis* were tested: ATCC 35984 (RP62A), ATCC 35983 and ATCC 12228 (ATCC, American Type Collection Culture, Manassas, VA, USA). In addition, *S. epidermidis* HAM 892 (isogenic mutant of RP62A) was tested [67]. The strains, stored at −80 °C in porous beads (Microbank, Pro-Lab Diagnostics, Round Rock, TX, USA), were inoculated in blood agar plates (OXOID LTD., Basingstoke, Hampshire, UK) and incubated at 37 °C for 24 h to obtain cultures. Subsequently, they were cultivated in Trypticase Soy Agar (TSA) or Trypticase Soy Broth (TSB) (BBL^TM^, BD, Becton, Dickinson and Company, Spark, NV, USA) according to assay.

The antimicrobial activity of SEEP was determined according to guidelines of Clinical and Laboratory Standard Institute, CLSI [68]. From overnight cultures in TSB incubated at 37 °C in a Memmert heater (Model 850, Memmert GmbH + Co. KG, Schwabach, Germany), the bacterial inoculums were prepared. Bacterial suspension was adjusted to 82% of transmittance at 492 nm wavelength by spectrophotometer (Helios epsilon Model, Thermo Scientific, Waltham, MA, USA). Then, different dilutions in TSB were used for each assay.

The minimal inhibitory concentration (MIC) was deteremined by dilution in agar (the reason being that the presence of SEEP visibly increased the turbidity of the broth medium.). Flasks with 20 mL of TSA were sterilized in autoclave (Presoclave-II, P. Selecta, S.A, Abrera, Barcelona, Spain). Different concentrations of propolis or alcohol, i.e., from 12% to 0.05%, were added to the flasks once cold. The inoculum was prepared by dilution to reach approximately 10^7^ colony-forming units for ml (cfu/mL). From these, 2 µL were collected to deposit ~10^4^ cfu/spot on agar. The plates were incubated at 37 °C for 24 h. The MIC was recorded as the lowest concentration of SEEP that completely inhibited visible bacterial growth in agar under suitable incubation conditions.

The minimal bactericidal concentrations (MBC) n was determined by using the 96-well plate microdilution (Greiner bio-one GmbH, Frickenhausen, Germany) method. The wells contained 100 µL of the different concentrations of propolis extract in TSB and 100 µL of the bacterial suspensions (10^6^ cfu/mL). The microplates were incubated for 24 h at 37 °C. After the incubation time, an aliquot of 50 µL was sub-cultured on TSA and incubated for 24–48 h at 37 °C. MBC was recorded as the lowest concentration at which no bacterial growth was observed. Three separate experiments by duplicate were conducted for each concentration of SEEP or alcohol tested.

The effect of SEEP subinhibitory concentrations (1/2, 1/4 and 1/8 of MIC) on the growth of the *S. epidermidis* strains was also evaluated. With that purpose, 5 µL of the bacterial suspensions (10^6^ cfu/mL) were cultured on TSA plates with the different subinhibitory concentrations of propolis extract and alcohol concentrations. After 24 h of incubation, bacterial growth was obtained as cfu/plate for each sub-MICs evaluated. Negative controls containing only TSA without inoculum and propolis-free positive controls were also performed. Triplicates were conducted for each of propolis extract concentration studied.

### 3.8. Statistical Study

The results of the propolis extract activity on growth were processed statistically by analysis of variance (one-way ANOVA) with the statistical program SPSS v22 (IBM SPSS Statistics, Chicago, IL, USA). The normal distribution of the mean of data for all concentrations and strains was previously checked using the Shapiro–Wilk normality test.

All data are presented as mean ± standard deviation of at least three independent experiments. The difference between the means was determined as significant at the level of *p* < 0.05.

## 4. Conclusions

The new Spanish ethanolic extract of propolis (SEEP) tested is a promising source of phenolic compounds, containing an exceptionally high number of flavonoids. This later finding certainly grants great quality to this new propolis, given the remarkable antioxidant and antimicrobial activities that have been attributed to this class of phenols. Moreover, a few new compounds have been, for the first time, identified in SEEP. Remarkably, these compounds (i.e., Vanillic acid, 1-Acetoxypinoresinol, *p*-HPEA-EA and 3,4-DHPEA-EA) are known to be also present in olive oil and greatly contribute to its health benefits. Additionally, relatively high amounts of ferulic acid and querecetin were distinguished, both known for their important therapeutic benefits. Finally, the sensibility of *S. epidermidis* at low SEEP concentrations, i.e., at sub-MICs, was reasonably high, revealing the potential use of SEEP as a natural drug in many therapeutic applications. New evidence is still needed to identify the bioactive compounds responsible for the activity of this new propolis and elucidate their mechanisms of action, which remain the basis for discovering of new natural antibacterial agents to face the rise in the antibiotic resistance of microorganisms.

## Figures and Tables

**Table 1:** Polyphenol and chemical compounds identified in SEEP by LC-MS and GC-MS. (RT: retention time, min; Mw: molecular weight; *m*/*z*: mass to charge ratio). The compounds highlighted in bold have been previously found in olive oil.

Compounds Identified by LC-MS				
RT	Proposed Structure	Formula	M_w_	*m*/*z*
**Flavonoids**				
1.9	3-Methoxynobiletin	C22 H24 O9	432.142	432.143
2.0	Nobiletin	C21 H22 O8	402.1315	402.132
2.0	Quercetin-dimethyl ether-*O*-rutinoside	C29 H34 O16	638.1847	638.1877
2.2	Quercetin-dimethyl ether-*O*-glucuronide	C22 H20 O13	492.0904	492.088
5.1	Cirsimaritin	C17H14 O6	314.079	314.0781
4.86	Epigallocatechin	C15 H14 O7	306.074	306.0754
6.4	Quercetin 3-*O*-rhamnosylrhamnosyl-glucoside	C33 H40 O20	756.2113	756.2096
6.4	Quercetin 3-*O*-rutinoside	C27 H30 O16	610.1534	610.1511
6.9	Kaempferol-3-*O*-rutinoside	C27 H30 O15	594.1585	594.1559
7.0	Isorhamnetin 3-*O*-glucoside7-*O*-rhamnoside	C28 H32 O16	624.169	624.1659
7.5	Quercetin 4′-*O*-glucoside	C21 H20 O12	464.0955	464.0935
7.7	Quercetin 3-*O*-glucuronide	C21 H18 O13	478.0747	478.0747
7.8	Luteolin 7-*O*-glucuronide	C21 H18 O12	462.0798	462.0801
7.8	Isorhamnetin-3-*O*-glucuronide	C21 H18 O12	462.0798	462.0775
9.1	Dihydroquercetin	C15 H12 O7	304.0583	304.0569
9.5	Quercetin-3-*O*-rhamnoside	C21 H20 O11	448.1006	448.0985
12.6	Apigenin 6-*C*-glucoside	C21 H20 O10	432.1056	432.1039
13.7	Naringenin	C15 H12 O5	272.0685	272.069
14.6	Hispidulin	C16 H12 O6	300.0634	300.0629
15.1	Daidzin	C21 H20 O9	416.1107	416.1089
15.2	Quercetin-dimethyl ether	C17 H14 O7	330.074	330.0725
15.3	Sakuranetin	C16 H14 O5	286.0841	286.0827
16.6	Kaempferol	C15 H10 O6	286.0477	286.0481
18.2	Chrysoeriol 7-*O*-glucoside	C22 H22 O11	462.1162	462.1139
20.8	Formononetin	C16 H12 O4	268.0736	268.0723
24.7	Hesperetin	C16 H14 O6	302.079	302.0776
29.8	Eriodictyol	C15 H12 O6	288.0634	288.0621
33.2	Rhamnetin	C16 H12 O7	316.0583	316.0588
33.2	7,3′,4′-Trihydroxyflavone	C15 H10 O5	270.0528	270.0534
41.4	Chrysin	C15 H10 O4	254.0579	254.0568
53.2	Caffeic acid phenylethyl ester (CAPE)	C17 H16 O4	284.1049	284.1035
56.4	Arbutin	C12 H16 O7	272.0896	272.0904
58.2	Chrysoeriol7-*O*-(6″-malonyl-apiosyl-glucoside)	C30 H32 O18	680.1589	680.1556
**Phenolic Acids**				
3.1	Caffeic acid 4-Oglucoside	C15 H18 O9	342.0951	342.0937
3.2	Hydroxycaffeic acid	C9 H8 O5	196.0372	196.0362
5.5	*p*-Coumaroyl tartaric acid	C13H12O8	296.0532	296.0521
6.4	**Ferulic acid**	**C10 H10 O4**	**194.0579**	**194.0584**
8.5	Vanillic acid *	**C8 H8 O4**	**168.0423**	**168.0422**
12.6	5-8′-Dehydrodiferulic acid	C20 H18 O8	386.1002	386.1007
15.5	Cinnamic acid	C9 H8 O2	148.0524	148.0518
19.0	*p*-Coumaric acid methyl ester	C10 H10 O3	178.063	178.0622
19.1	Hydroxyphenyl propionate	C9 H10 O3	166.063	166.0622
21.9	*p*-Coumaric acid isoprenyl ester	C14 H14 O3	230.0943	230.0932
27.1	*p*-Coumaric acid ethyl ester	C11 H12 O3	192.0786	192.0779
30.6	Cinnamyliden acetic acid	C11 H10 O2	174.0681	174.0674
41.4	*p*-Coumaroyl tyrosine	C18 H17 N O5	327.1107	327.1102
52.4	Caffeic acid cinnamyl ester	C18 H16 O4	296.1049	296.1035
60.7	Cinnamoyl glucose	C15 H18 O7	310.1053	310.1052
**Lignans**				
15.1	Episesaminol	C20 H18 O7	370.1053	370.1035
18.2	1-Acetoxypinoresinol *	**C22 H24 O8**	**416.1471**	**416.1452**
**Others Polyphenols**				
4.2	Sinapaldehyde	C11 H12 O4	208.0736	208.0726
7.0	*p*-HPEA-EA *	**C19 H22 O7**	**362.1366**	**362.1349**
67.3	Demethoxycurcumin	C20 H18 O5	338.1154	338.1138
7.8	Coumarin	C9 H6 O2	146.0368	146.0363
34.5	3,4-DHPEA-EDA *	**C17 H20 O6**	**320.126**	**320.1245**
**Compounds Identified by GC-MS**				
**RT (min)**		**Match Result**	**Compound**	
8.486		937	Benzyl Alcohol	
9.942		947	Phenylethyl Alcohol	
10.683		929	Benzoic acid	
12.583		836	Benzenepropanal	
**13.621**		**938**	**Vanillin**	

* For the first time in propolis. ***p*-HPEA-EA**, *p*-HPEA-Elenolic acid mono-Aldehyde; Ligstroside-aglycone mono-aldehyde; (Ligstroside-aglycone major form); **3,4-DHPEA-EDA**, dialdehydic form of elenolic acid linked to hydroxytyrosol; 3,4-DHPEA-Elenolic acid Di-Aldehyde; Oleuropein-aglycone di-aldehyde; (Decarboxymethyl oleuropein-aglycone major form).

**Table 2:** Calibration curves for marker compounds at concentrations ranging from 5 to 300 (μg/mL). R^2^ represents the goodness of the fit.

Compound	Range	Calibration Curve	R^2^	Quantity (ppm)
Vanillic acid	5–200	y = 23263x − 58213	0.9991	5.2
Trans-ferulic acid	10–300	y = 120462x + 627419	0.9974	250
Quercetin	10–200	y = 196425x + 2 × 10^6^	0.9817	23.5

**Table 3:** Concentrations of different inorganic compounds found in SEEP determined by Wavelength Dispersive X-Ray Fluorescence.

Formula	Z	Concentration	Line 1	Calc. Concentration	Stat. Error
K_2_O	19	135 ppm	K KA1-HR-Tr	0.0134	4.38%
P_2_O_5_	15	44.8 ppm	P KA1-HR-Tr	0.004	19.20%
ZnO	30	10.9 ppm	Zn KA1-HR-Tr	0.001	6.93%
CuO	29	5.42 ppm	Cu KA1-HR-Tr	0.001	14.60%

**Table 4:** Antibacterial activity (MIC and MBC or range) of ethanolic extract of propolis from different geographical origins against *Staphylococcus epidermidis* strains.

*Staphylococcus epidermidis* (n)	Propolis Origin	MIC/Range (μg/mL)	MBC/Range (μg/mL)	Methodology	References
4	Extremadura (Southwest of Spain)	240	480	MIC—Agar dilution MBC—Microdilution in broth and subcultured on agar	Present work
2	Poplar Type propolis (France)	>100	-	Agar dilution	[48]
1	Lyon (France)	3000	-	Agar dilution	[49]
1	Germany, Ireland and Czech Republic	600	1200	MIC—Microdilution in broth MBC—Subcultured on blood agar	[56]
63	Italy	620–2500	-	Agar dilution	[50]
11	Poland	780–1560	-	Microdilution in broth	[57]
2	Serbia	780–6300	-	Agar dilution	[51]
1	Cretan propolis (Greece)	50	-	Microdilution in broth	[46]
1	Greek propolis (Northwest Greece)	750	-	Microdilution in broth	[58]
1	Anatolian propolis (Turkey)	8–32 *	-	Macrodilution in broth	[47]
1	Cameroon and Congo propolis (Africa)	10850–20000 *	-	Microdilution in broth	[59]
2	Brazil	770–880	1750–1920	MIC—Microdilution in broth MBC—TCC staining lecture and subcultured on NB agar	[60]
1	Brazil	10700	-	Macrodilution tube	[61]
1	Huasteca Potosina (México)	-	1870–30000	MBC—Macrodilution tube and subcultured on agar	[62]

MIC: minimum inhibitory concentration, MBC: minimum bactericidal concentration, (-) No data, (*) The range was calculated from diferent propolis samples studied.

## Abbreviations

ANOVA	Analysis of variance
ATCC	American Type Culture Collection
Cfu	Colony formation unit
CLSI	Clinical and Laboratory Standard Institute
GAE	Gallic acid equivalents
GC-MS	Gas chromatography coupled to mass spectrometry
LC-MS	Liquid chromatographic mass spectrometry
LOD	Limit of detection
LOQ	Limit of quantification
MBC	Minimum bactericidal concentrations
MIC	Minimum inhibitory concentrations
*p*-HPEA-EA	*p*-HPEA-Elenolic acid mono-Aldehyde
QE	Quercetin equivalent
*S. epidermidis*	*Staphylococcus epidermidis*
SEEP	Spanish ethanolic extracts of propolis
TFC	Total flavonoid content
TPC	Total polyphenol content
TSB	Trypticase Soy Broth
WDXRF	Wavelength dispersive X ray fluorescence
3,4-DHPEA-EDA	dialdehydic form of elenolic acid linked to hydroxytyrosol
